# Primary Graft Dysfunction After Lung Transplantation: A Temporal Classification and Machine Learning Clustering

**DOI:** 10.1097/TXD.0000000000001984

**Published:** 2026-07-20

**Authors:** Balin Özsoy, Bieke Vercauteren, Jan Van Slambrouck, Cedric Vanluyten, Annalisa Barbarossa, Xin Jin, Dirk E. Van Raemdonck, Julia Dmitrieva, Anwar Khan, Xander Jacquemyn, Saskia Bos, Robin Vos, Bart N. Vanaudenaerde, Marianne S. Carlon, Jean-Marie Aerts, Peter Carmeliet, Laurens J. Ceulemans

**Affiliations:** 1 Department of Thoracic Surgery, University Hospitals Leuven, Leuven, Belgium.; 2 Laboratory of Respiratory Diseases and Thoracic Surgery (BREATHE), Department of Chronic Diseases and Metabolism, KU Leuven, Leuven, Belgium.; 3 Laboratory of Angiogenesis and Vascular Metabolism, Department of Oncology, Center for Cancer Biology, VIB, KU Leuven, Leuven, Belgium.; 4 Division Animal and Human Health Engineering, Department of Biosystems, KU Leuven, Leuven, Belgium.; 5 Department of Respiratory Diseases, University Hospitals Leuven, Leuven, Belgium.; 6 Center for Biotechnology, Khalifa University of Science and Technology, Abu Dhabi, United Arab Emirates.

## Abstract

**Background.:**

Primary graft dysfunction (PGD) is a major cause of morbidity and mortality after lung transplantation (LTx). PGD is graded at static time points, limiting insight into its temporal dynamics. Statistical risk-factor analysis may overlook the multifactorial complexity of PGD. Machine learning (ML) may address this but is constrained by small sample sizes. We aim to introduce a temporal PGD classification, perform ML-based clustering and overcome sample-size limitations by generating synthetic patient data.

**Methods.:**

A prospectively collected database of 794 LTx at University Hospitals Leuven (Belgium) from January 2012 to November 2023 was analyzed. Recipients were classified into 4 temporal PGD phenotypes: no, early, late, and persistent PGD. Unsupervised ensemble *k*-means clustering was used to group patients based on 30 clinical variables. To generate synthetic data, a Wasserstein Generative Adversarial Network with Gradient Penalty was trained.

**Results.:**

Six hundred ninety primary double LTx cases were included. Temporal PGD phenotypes showed significantly different 5-y survival (log-rank test: *P < *0.001). *K*-means clustering revealed stable patient subgroups, with *k = *5 and *k = *9 identified as optimal cluster numbers. Key features affecting clustering included preoperative intensive care unit stay and postoperative chest x-rays. The synthetic data set preserved the correlation structure of the original data, and the combined data had similar clustering behavior, with optimal clustering at *k = *5, *k = *7, and *k = *9.

**Conclusions.:**

This study demonstrates the potential of temporal PGD classification to better understand how the dynamic character of PGD affects survival. ML techniques, including unsupervised clustering and synthetic data generation, could be promising strategies to unravel complex interactions between clinical factors and overcome sample-size limitations.

## INTRODUCTION

Primary graft dysfunction (PGD) is an umbrella term for the syndrome of acute lung injury in the first 72 h after lung transplantation (LTx), encompassing a range of pathophysiological processes.^1,2^ Severe PGD occurs in 30% of patients within the first 72 h after LTx and is an important risk factor for morbidity and mortality.^3^ The current grading system for PGD was introduced to unify the definition between different centers. The 2016 consensus from the International Society for Heart and Lung Transplantation (ISHLT) evaluates chest x-ray findings and PaO_2_/FiO_2_ ratios at fixed time points (0, 24, 48, 72 h) and gives scores ranging from PGD0 to PGD3.^4^ Clinical LTx studies generally use static phenotypes of PGD3 at or within 72 h to group patients.^5,6^ However, this approach fails to capture the dynamic nature of PGD. Temporal phenotypes of PGD have been described, such as “early transient” and “late-onset,” but the relationship between these phenotypes and outcome remains overlooked.^1,7-9^

Several risk factors that contribute to PGD have been identified, for example, donor smoking, transplant indication, anastomosis time, and so on.^10-12^ Traditionally, determining risk factors and predicting survival rely on statistical methods that fail to account for complex interactions among multiple clinical factors.^13,14^ In recent years, machine learning (ML) has emerged as a promising tool for predicting outcomes, mostly based on supervised learning.^15-22^

Beyond prediction, ML may also offer a data-driven approach to refine classification. Unsupervised learning focuses on identifying patterns within the data.^23,24^ This underexplored approach could cluster patients into separate groups and identify interdependent risk factors by determining the relevance of clinical factors (also called “features” in ML language). ML algorithms depend on a high quantity and quality of input. In contexts with a limited sample size, such as LTx, biases from imbalanced data can affect generalizability.^25,26^ Generation of synthetic data, where the model learns patterns of the original data set to produce an artificial data set, can overcome this issue.^27^

Considering the ongoing challenges in understanding PGD phenotypes and risk factors, our objective is 3-fold: first, we aim to develop a temporal classification of PGD capturing the dynamic evolution rather than relying on static, single time point grades and study its relation to survival. Second, we propose an unsupervised ML approach to cluster patients into risk groups and understand the determining factors for clustering. Third, we apply an ML algorithm to create synthetic patient data to increase the sample size and validate the combined original and synthetic data set using the unsupervised ML model.

## PATIENTS AND METHODS

### Study Population

We performed a single-center retrospective analysis of a prospectively collected database of all LTx cases at University Hospitals Leuven, Belgium, from January 1, 2012, to November 30, 2023, with a follow-up until February 24, 2024. Patients were excluded if they died within 72 h after LTx, had combined organ transplants, single or lobar LTx, retransplants, or had ungradable or missing PGD data. The study was approved by the ethics committee of UZ/KU Leuven (S67904). Patients provided written informed consent.

### Data Collection

Donor characteristics included sex, age, body mass index (BMI), cause of death, donation type (brain death or circulatory death), ventilation time, smoking, chest x-rays, and last PaO_2_/FiO_2_ ratio. Recipient characteristics included sex, age, BMI, transplant indication, waitlist time, previous thoracic surgery (wedge biopsies and anatomical resections), preoperative intensive care unit (ICU)/hospital stay, extracorporeal membrane oxygenation bridging, and mechanical ventilation. The preservation method was also included. Intraoperative variables included total ischemia time (longest of 2 lungs), implantation time (longest of 2 lungs), and extracorporeal life support (ECLS). Postoperative variables included intubation time, need for extracorporeal membrane oxygenation support, chest x-ray findings (bilateral alveolar infiltrates in x-rays immediately after operation [0 h] and daily for 3 d [24, 48, 72 h]), and PaO_2_/FiO_2_ ratios within 72 h, length of ICU/hospital stay, surgical reinterventions, and 1- and 5-y patient survival.

### Temporal PGD Classification

**FIGURE 1.**
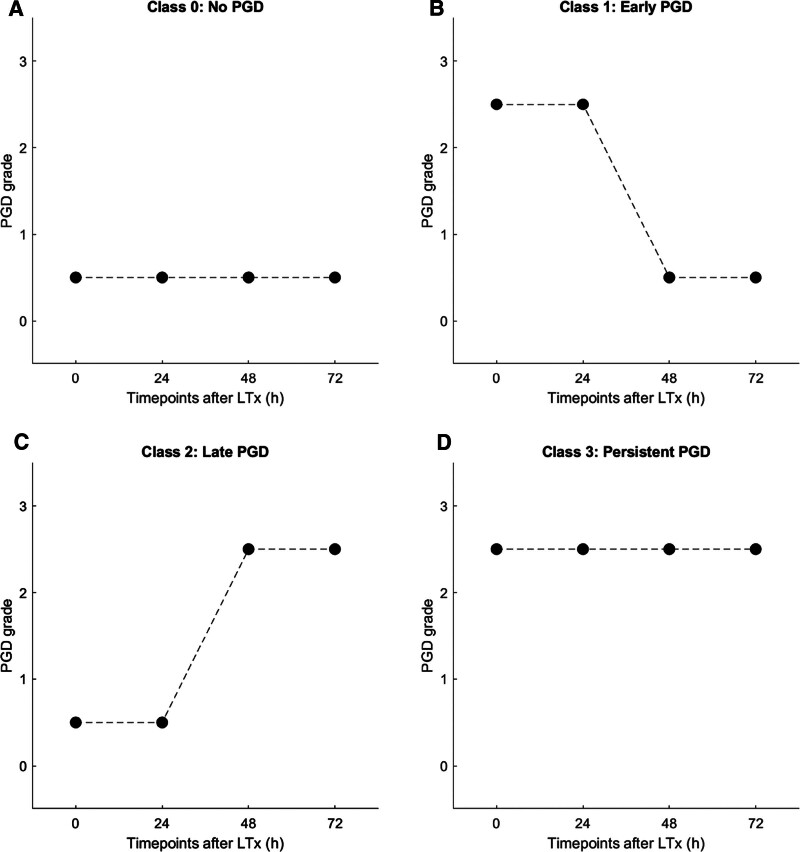
Representation of temporal PGD classification based on the evolution of PGD grades within the first 72 h after LTx: no PGD (A), early PGD (B), late PGD (C), and persistent PGD (D). LTx, lung transplantation; PGD, primary graft dysfunction.

PGD severity was graded according to the 2016 ISHLT guidelines.^4^ Chest x-rays were independently evaluated by 2 readers. A temporal classification of 4 classes was developed on the basis of clinical observation^28^ (Figure 1): (1) no PGD: PGD grade 0 or 1 at all time points; (2) early PGD: PGD grade ≥2 occurring at either 0 h or 24 h with a grade <2 at 72 h; (3) late PGD: PGD grade ≥2 with onset occurring at either 48 h or 72 h; and (4) persistent PGD: PGD grade ≥2 at least at 2 time points, including at 0 h or 24 h and at 72 h.

### Statistical Analysis

Patient demographics were summarized as median (interquartile range) or frequency (%). Survival was analyzed by Kaplan-Meier and reported with 95% confidence intervals. The effect of temporal PGD classes on 5-ysurvival was evaluated using a multivariable Cox proportional hazards model, adjusted for clinically relevant covariates. Analyses were conducted with R version 4.4.2 (R Foundation for Statistical Computing) using RStudio (RStudio, PBC) and Prism version 10.4.2 (GraphPad, USA).

### *K*-means Clustering

K-means clustering (meaning: grouping data in a number [* = k*] of clusters) is an unsupervised ML method that groups data points based on feature similarity. We complied with TRIPOD+AI guidelines where applicable to ensure transparent reporting of our ML approach (**Table S1, SDC,** https://links.lww.com/TXD/A884; further explained in **Supplemental Methods, SDC,** https://links.lww.com/TXD/A884).^29^ Clustering was performed on a preprocessed data set based on selected clinical features (Table 1) using an ensemble consensus approach. The number of clusters, *k*, varied from 2 to 25. Cluster quality was evaluated using silhouette scores and within-cluster sum of squares (WCSS). Spread of clusters was analyzed to calculate feature-wise range and SD (meaning: the range and SD between the centroids [centers of clusters]). To visualize, principal component analysis (PCA) and Sankey diagrams were used.^30^ Clustering and analyses were conducted in MATLAB R2024b (MathWorks Inc.).

**TABLE 1.** Features included in the unsupervised clustering

Features
Donor characteristics
Donor type
DBD
DCD
Age, y
Sex
Male
Female
BMI, kg/m^2^
Mode of death
CVA
Head trauma
Other
Ventilation, h
Smoking
Smoking
Non-smoking
Smoking data missing
Radiological findings
Normal
Abnormal
Radiological findings missing
PaO_2_/FiO_2_ ratio, mm Hg
Recipient characteristics
Age, y
Sex
Male
Female
BMI, kg/m^2^
Indication for transplant
COPD
Interstitial lung disease
Cystic fibrosis
Other
Previous thoracic surgery
Yes
No
Preoperative hospital stay
Yes
No
Preoperative ICU stay
Yes
No
Preoperative ventilation
Invasive
Noninvasive
No
ECMO bridging
Yes
No
Preservation
Ice storage
Ex vivo lung perfusion
Controlled hypothermic storage
Intraoperative
Total ischemia time: longest, min
Implantation time: longest, min
Intraoperative ECLS
Yes
No
Postoperative
Chest x-ray at 0 h
Edema
No edema
Chest x-ray at 24 h
Edema
No edema
Chest x-ray at 48 h
Edema
No edema
Chest x-ray at 72 h
Edema
No edema
PaO_2_/FiO_2_ ratio at 0 h, mm Hg
PaO_2_/FiO_2_ ratio at 24 h, mm Hg
PaO_2_/FiO_2_ ratio at 48 h, mm Hg
PaO_2_/FiO_2_ ratio at 72 h, mm Hg

Continuous features have their unit between brackets; all other features are categorical. Donor type was grouped as DCD or DBD. Cause of death was grouped as cerebrovascular accident, head trauma, or other. Radiological findings were grouped as abnormal or normal. Indication for transplant was grouped as chronic obstructive pulmonary disease, interstitial lung disease, cystic fibrosis, or other. Preoperative ventilation was evaluated as invasive and noninvasive. Preservation was grouped as ice storage, controlled hypothermic storage, and ex vivo lung perfusion. For categorical features with missing data, a missing-data category was added.

BMI, body mass index; COPD, chronic obstructive pulmonary disease; CVA, cerebrovascular accident; DBD, donation after brain death; DCD, donation after circulatory death; ECLS, extracorporeal life support; ECMO, extracorporeal membrane oxygenation; ICU, intensive care unit; PaO_2_/FiO_2_, ratio of arterial oxygen partial pressure to fractional inspired oxygen.

### Synthetic Data Generation

A Wasserstein Generative Adversarial Network with Gradient Penalty (WGAN-GP) was used to generate synthetic data, consisting of 2 neural networks (explained in **Supplemental Methods, SDC,** https://links.lww.com/TXD/A884). The synthetic data set reached the same size as the original data set to have a balanced downstream comparison.

Because WGAN-GP is traditionally used as an image generator, the similarity between the original and synthetic data sets was assessed visually using various plots.^31^ Correlation matrices were presented as heatmaps and compared using a difference matrix. To evaluate the downstream utility of the combined data set, it was used as input for clustering with the previously explained consensus method. The WGAN-GP was implemented in Python version 3.12.4 (Python Software Foundation) using Visual Studio Code (Microsoft Corporation), and clustering was performed in MATLAB R2024b (The MathWorks Inc.).

## RESULTS

### Cohort Characteristics and Outcomes

**FIGURE 2.**
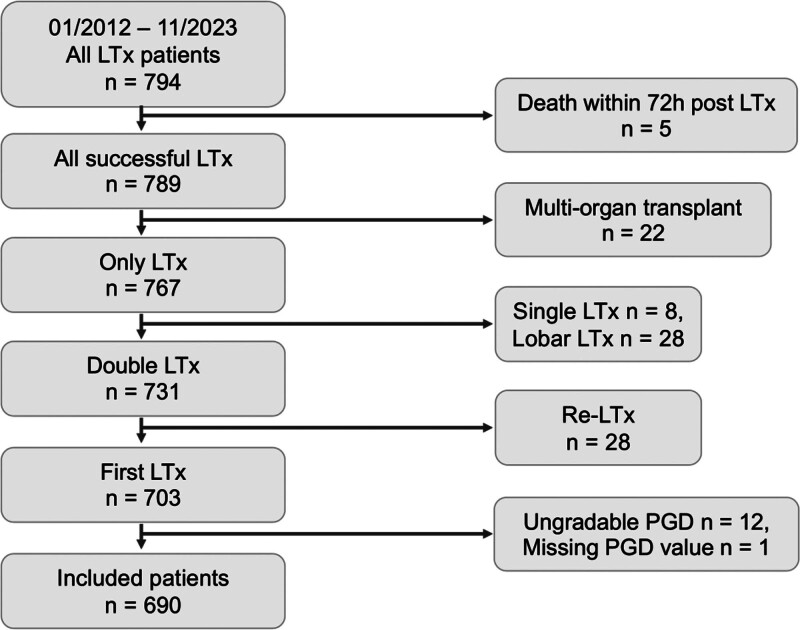
Flowchart diagram of the study cohort. LTx, lung transplantation; PGD, primary graft dysfunction.

Six hundred ninety of 794 LTx cases in the study period were included (Figure 2). Donor and recipient characteristics, preservation method, and intraoperative and postoperative variables are summarized in Table 2. The donor age was 54 (42–63) y, and 317 (46%) donors were women. One hundred ninety-two donors (28%) were donors after circulatory death. The main cause of death was cerebrovascular accident (n* = *324; 47%). Recipient age was 59 (52–62) y, and 334 (48%) recipients were women. Recipients predominantly had a chronic obstructive pulmonary disease diagnosis (n* = *398; 58%) and 131 (19%) had undergone previous thoracic surgery. Ice storage was the most used preservation method (n* = *647; 94%). ECLS was used intraoperatively in 128 cases (19%). Posttransplant ICU and hospital stay were 6 (4–12) and 28 (23–40) d, respectively. 206 patients (30%) developed PGD3 within 72 h, and 81 (12%) had PGD3 at 72 h (**Figure S1, SDC,** https://links.lww.com/TXD/A884). One-year and 5-y survival was 92% (89%–94%) and 77% (74%–81%), respectively.

**TABLE 2.** Donor, recipient, preservation, intraoperative, and postoperative characteristics and outcomes

	All patients (N = 690)
Donor characteristics	
Female sex	317 (46%)
Age, y	54 (42–63)
BMI, kg/m^2^	24.67 (22.49–27.68)
Donor type	
DCD	192 (28%)
Cause of death	
Cerebrovascular accident	324 (47%)
Head trauma	106 (15%)
Other	260 (38%)
Ventilation, h	72 (44–127.88)
Smoking (N = 644 available)	216 (34%)
Last PaO_2_/FiO_2_ ratio (N = 669 available)	440 (384–498)
Abnormal x-ray findings (N = 588 available)	248 (42%)
Recipient characteristics	
Female sex	334 (48%)
Age, y	59 (52–62)
BMI, kg/m^2^	23.00 (19.47–26.33)
Indication for transplant	
COPD	398 (58%)
Interstitial lung disease	171 (25%)
Cystic fibrosis	73 (11%)
Other	48 (7%)
Waitlist time, d	241 (89.25–441.75)
Previous thoracic surgery	131 (19%)
Preoperative hospital stay	76 (11%)
Preoperative ICU stay	50 (7%)
ECMO bridging	24 (3%)
Preoperative mechanical ventilation	
Invasive	24 (3%)
Noninvasive	8 (1%)
Preservation	
Ice storage	647 (94%)
Ex vivo lung perfusion	24 (4%)
Controlled hypothermic storage	14 (2%)
Intraoperative	
Total ischemia time: longest, min (N = 686 available)	487 (421.25–554.75)
Implantation time: longest, min (N = 687 available)	73 (65–84)
Intraoperative ECLS	128 (19%)
Postoperative	
Intubation, d (N = 413 available)	1.82 (1.13–3.92)
ECMO	33 (5%)
PGD3 within 72 h	206 (30%)
PGD at 72 h	
PGD0	279 (40%)
PGD1	199 (29%)
PGD2	131 (19%)
PGD3	81 (12%)
ICU stay, d	6 (4–12)
Hospital stay, d	28 (23–40)
Reintervention (within 90 d) (N = 623 available)	146 (23%)
1-y survival	92% (89%–94%)
5-y survival	77% (74%–81%)

BMI, body mass index; COPD, chronic obstructive pulmonary disease; DCD, donation after circulatory death; ECLS, extracorporeal life support; ECMO, extracorporeal membrane oxygenation; ICU, intensive care unit; PaO_2_/FiO_2_, ratio of arterial oxygen partial pressure to fractional inspired oxygen; PGD, primary graft dysfunction.

### Temporal PGD Classification

**FIGURE 3.**
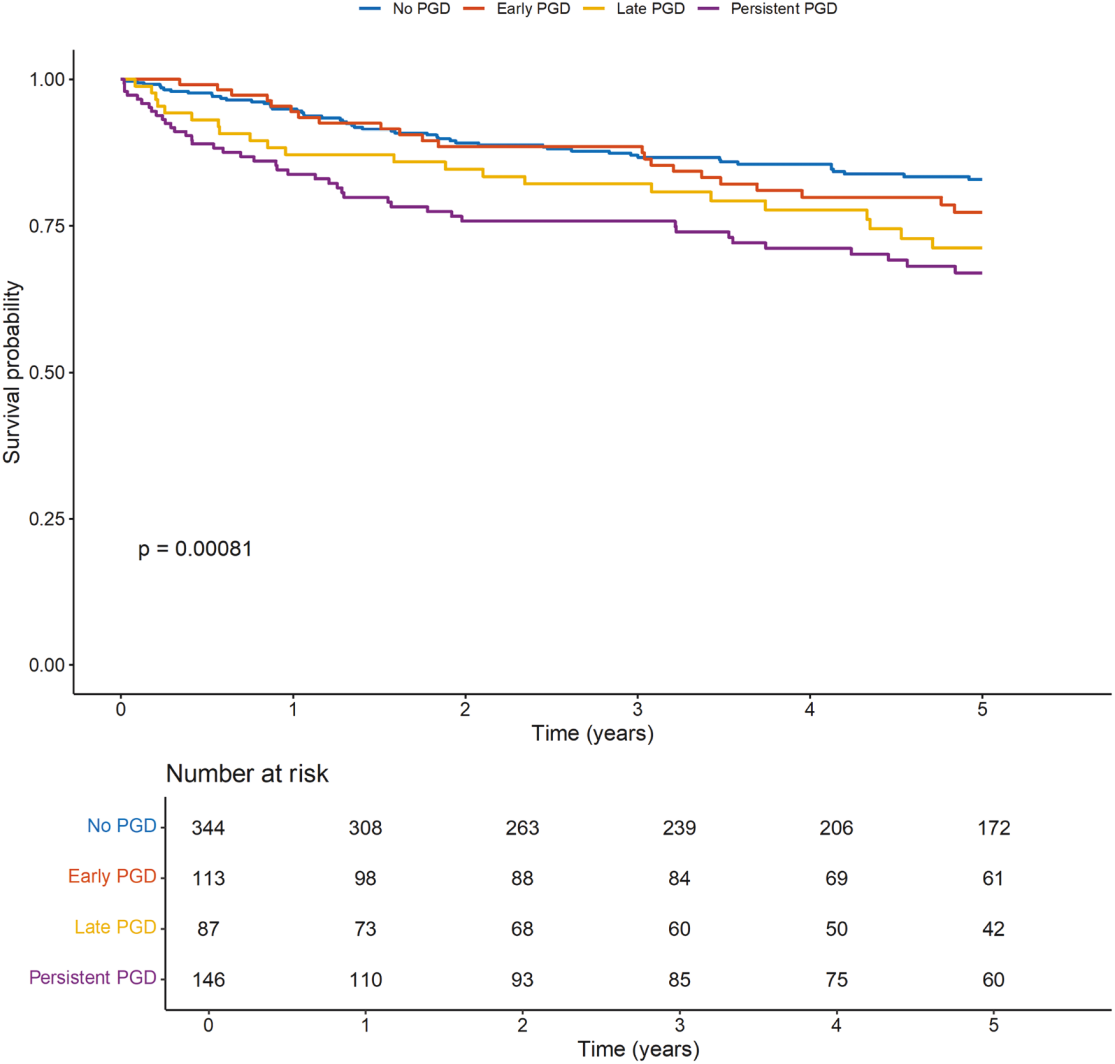
Kaplan-Meier estimates of the 4 subgroups of temporal PGD classification, with numbers at risk. PGD, primary graft dysfunction.

The patients were classified into 4 temporal PGD classes: no PGD (n* = *344; 50%), early PGD (n* = *113, 16%), late PGD (n* = *87; 13%), and persistent PGD (n* = *146; 21%). Kaplan-Meier survival analysis revealed a significant difference in 5-y survival rates among temporal PGD classes (*P = *0.00081; Figure 3). Patients without PGD had the highest 5-y survival (83%, 79%–87%), whereas patients with persistent PGD had the poorest survival (67%, 60%–76%). Distinct separation could also be observed between early PGD (77%, 69%–86%) and late PGD (71%, 62%–82%) phenotypes, the latter resulting in lower survival. On multivariable Cox analysis, Persistent PGD independently predicted 5-y survival (hazard ratio, 1.84; 95% confidence interval, 1.17-2.89; *P = *0.008; **Table S2, SDC,** https://links.lww.com/TXD/A884).

### Clustering of the Original Patient Data Set

A total of 30 clinical features were incorporated into the ensemble clustering algorithm (Table 1). To determine the optimal number of groups, clustering was performed from *k = *2 to *k = *25.

**FIGURE 4.**
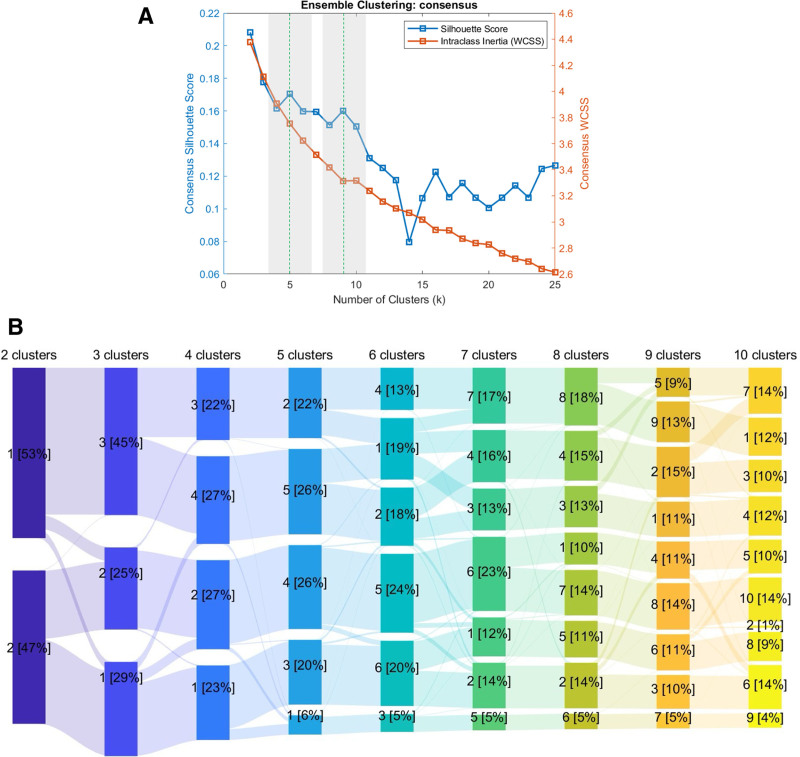
Clustering evaluation across 2 to 25 clusters (*k*) for the original patient data set. A, Relationship between the number of clusters (*k*) and 2 clustering performance metrics: silhouette score (left y-axis, blue) and WCSS (right y-axis, orange). Silhouette score reflects how well separated the clusters are, where higher values indicate more distinct clusters. WCSS quantifies the compactness of clusters, where lower values indicate tighter groupings. Preferably, the clusters should be well separated and compact. Dashed green lines indicate optimal clustering options, at *k* = 5 and *k* = 9. Gray zone indicates the region of the elbow point, where the slope of the WCSS curve flattens. B, Sankey diagram showing the redistribution of patient assignments across an increasing number of clusters. Each vertical band represents a cluster at a specific value of *k*. The width of each band corresponds to the proportion of patients within that cluster. Flows between bands indicate how individual patients are reassigned across clusters as *k* increases, where the thickness represents the number of patients. Stable flows, where patient groups remain cohesive across adjacent *k*, suggest robust subgroup structure. WCSS, within-cluster sum of squares.

Silhouette scores and WCSS for clusters are presented in Figure 4A. Based on the identification of elbow points in the WCSS curve (compactness of clusters) and peaks in silhouette scores (separation of clusters), both *k = *5 and *k = *9 are promising options. Sankey diagram (Figure 4B) visualizes patient transitions across clusters. Many patient groupings remain stable and conserved across *k*-values.

**FIGURE 5.**
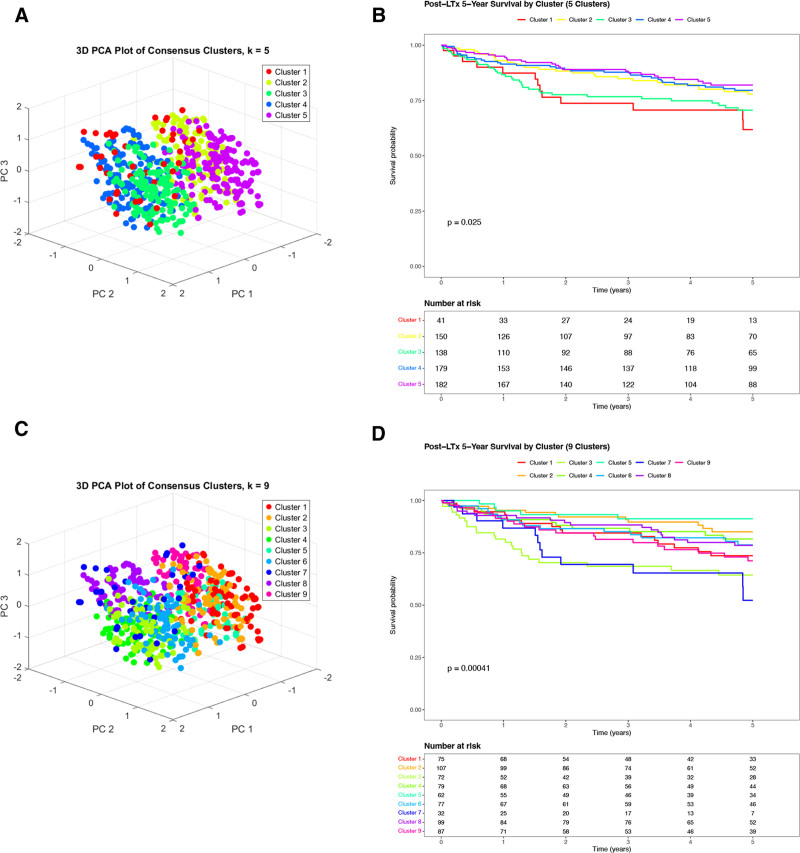
PCA plots and Kaplan-Meier survival curves for *k* = 5 and *k* = 9 clusters for the original data set. The clusters are indicated using the same colors in the PCA plots and corresponding survival curves. A, Three-dimensional PCA plot for visualizing the separation and spatial distribution of clusters, for *k* = 5. High-dimensional patient data is projected into 3 PCs, with percentage of variance in brackets. Color-coded points indicating the cluster assignment of each patient. B, Kaplan-Meier estimates for *k* = 5 with numbers at risk. C, Three-dimensional PCA plot for *k* = 9. D, Kaplan-Meier estimates for *k* = 9 with numbers at risk. LTx, lung transplantation; PC, principal component; PCA, PC analysis.

Based on these results, the *k = *5 and *k = *9 configurations were considered suitable for further exploration and were visualized using 3-dimensional PCA, where each dot represents a patient, and also compared in terms of survival (Figure 5). Color-coded PCA plots for *k = *5 show a clear distinction between groups, with 4 main clusters and a cluster that separates above (cluster 1 [in red]; Figure 5A), which included cases with more preoperative ICU stay, longer implantation time, and more intraoperative ECLS (data not shown). This cluster also matches with cluster 7 in *k = *9 (Figure 4B), showing similar trends in the Kaplan-Meier curves (Figure 5B and D) and revealing the worst survival.

Feature importance was assessed on the basis of cluster centroid range and SD to determine which features affect clustering. Feature importance tables (**Table S3, SDC,** https://links.lww.com/TXD/A884) demonstrate that preoperative ICU stay, postoperative chest x-rays, and donor and recipient sex represent the most discriminative features, both for *k = *5 and *k = *9. Donor smoking is one of the most important features for *k = *9, whereas for *k = *5, it is of intermediate importance. Less important features for both configurations are donor BMI, PaO_2_/FiO_2_, ventilation, and preservation method.

### Synthetic Data Generation

The WGAN-GP model demonstrated convergence >8000 training epochs. Both generator and critic losses became steady after an initial period of rapid fluctuation (**Figure S2, SDC,** https://links.lww.com/TXD/A884). Generator loss exhibited a gradual decrease and plateaued between –0.5 and –0.3, whereas critic loss converged around –0.5, consistent with theoretical expectations for WGAN-GP.

**FIGURE 6.**
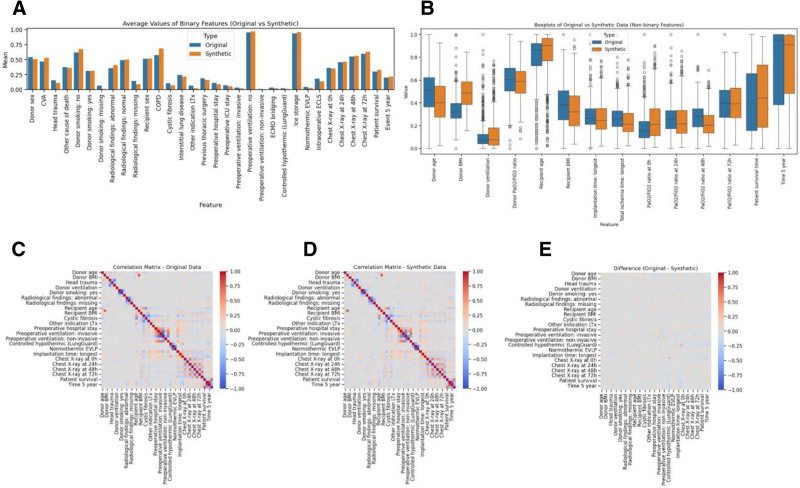
Evaluation of the synthetic data set. A, Boxplots of nonbinary features in the original (blue) and synthetic (orange) data sets showing medians, interquartile ranges, and outliers. B, The mean values of binary features for both original and synthetic data sets. The correlation matrices for original (C) and synthetic (D) data, and the difference matrix (E); measuring pairwise correlation coefficients between features. The original data matrix (C) reveals a complex structure of both positive and negative correlations. The synthetic data matrix (D) largely preserves these correlation patterns, also shown as a subtraction in (E). The features on the axes are indicative, whereas the heatmap includes all numerical and categorical features. BMI, body mass index; COPD, chronic obstructive pulmonary disease; CVA, cerebrovascular accident; DBD, donation after brain death; DCD, donation after circulatory death; ECLS, extracorporeal life support; ECMO, extracorporeal membrane oxygenation; EVLP, ex vivo lung perfusion; ICU, intensive care unit; LTx, lung transplantation; PaO_2_/FiO_2_, ratio of arterial oxygen partial pressure to fractional inspired oxygen.

To assess synthetic data, multiple visual comparisons were performed. Box plots of normalized original versus synthetic numerical features (Figure 6A) revealed similar distributions across features. The average values of binary features are shown in Figure 6B. Correlation heatmaps comparing original and synthetic data sets (Figure 6C and D) showed a high degree of structural similarity in feature interdependencies (eg, the link between donor and recipient sex due to sex matching, and preoperative ventilation and hospital stay). This was confirmed by the difference matrix (Figure 6E).

**FIGURE 7.**
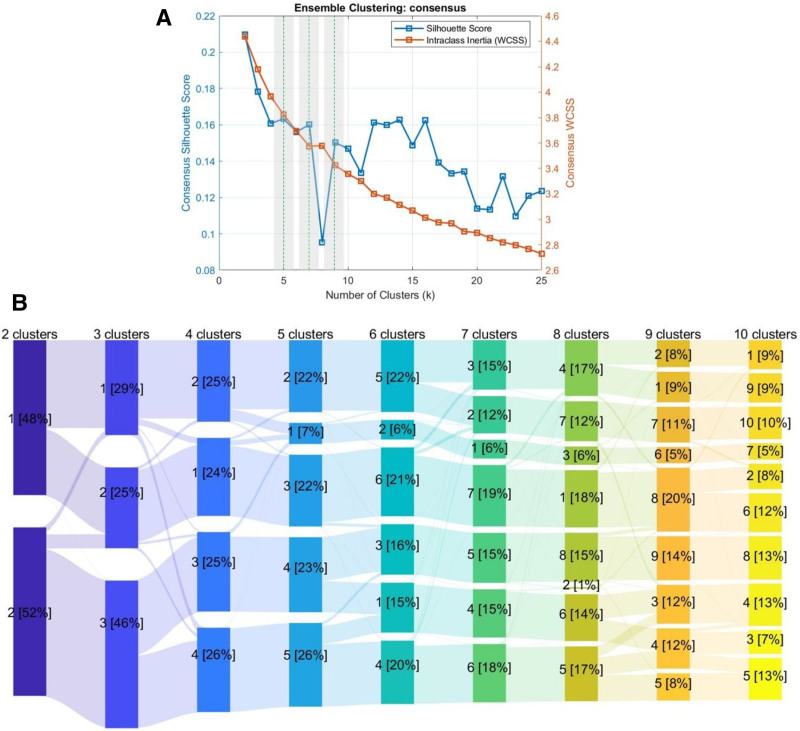
Clustering evaluation across 2–25 clusters (*k*) for the combined data set of original and synthetic data. A, Relationship between the number of clusters (*k*) and 2 clustering performance metrics: silhouette score (left y-axis, blue) and WCSS (right y-axis, orange). Silhouette score reflects how well separated the clusters are, where higher values indicate more distinct clusters. WCSS quantifies the compactness of clusters, where lower values indicate tighter groupings. Preferably, the clusters should be well separated and compact. Dashed green lines indicate optimal clustering options, at *k* = 5, *k* = 7, and *k* = 9. *k* = 8 appears as a local outlier because it reflects an unstable and potentially unnatural split: it produces one very small cluster and 7 larger clusters, resulting in a much lower consensus silhouette score, although WCSS continues to decrease as expected with increasing k. B, Sankey diagram showing the redistribution of patient assignments across an increasing number of clusters. Each vertical band represents a cluster at a specific value of *k*. The width of each band corresponds to the proportion of patients within that cluster. Flows between bands indicate how individual patients are reassigned across clusters as *k* increases, where the thickness represents the number of patients. Stable flows, where patient groups remain cohesive across adjacent *k*, suggest robust subgroup structure. WCSS, within-cluster sum of square.

**FIGURE 8.**
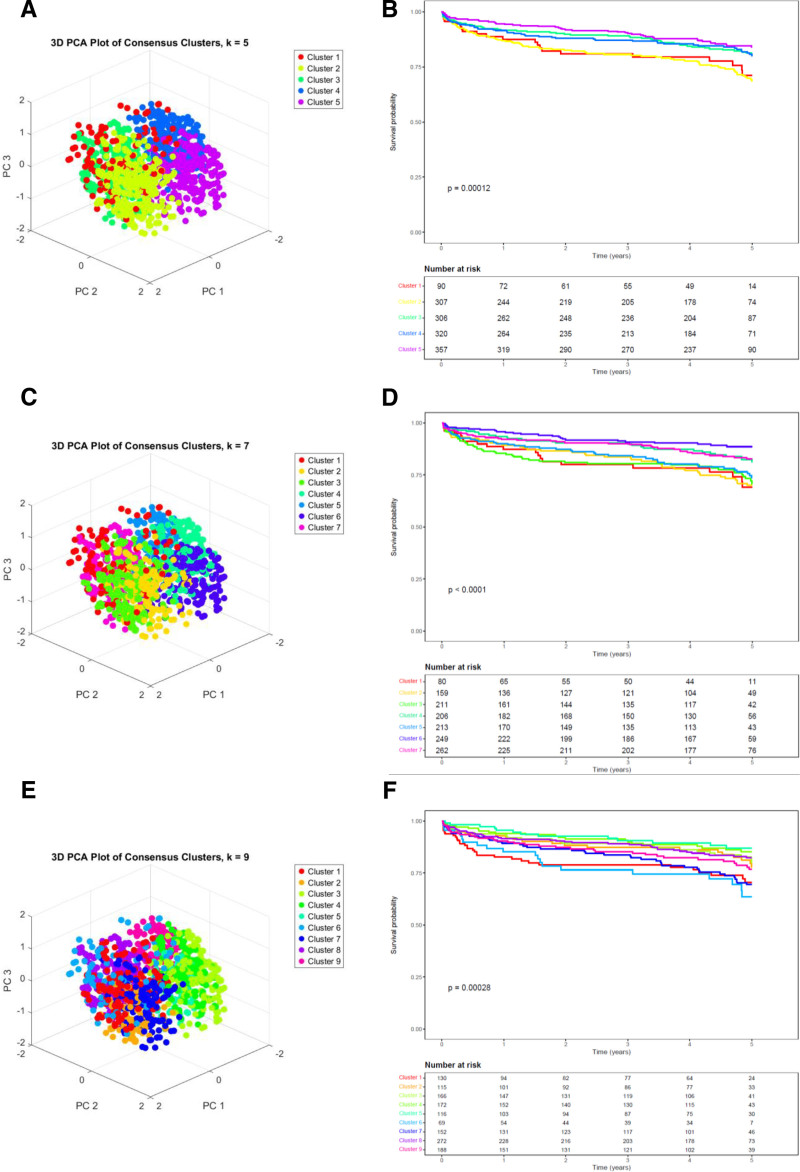
PCA plots and Kaplan-Meier survival curves for *k* = 5, *k* = 7, and *k* = 9—clusters for the combined data set of original and synthetic data. The clusters are indicated using the same colors in the PCA plots and corresponding survival curves. A, Three-dimensional PCA plot for visualizing the separation and spatial distribution of clusters, for *k* = 5. High-dimensional patient data are projected into 3 principal components, with the percentage of variance in brackets. Color-coded points indicating the cluster assignment of each patient. B, Kaplan-Meier estimates for *k* = 5 with numbers at risk. C, Three-dimensional PCA plot for *k* = 7. D, Kaplan-Meier estimates for *k* = 7 with numbers at risk. E, Three-dimensional PCA plot for *k* = 9. F, Kaplan-Meier estimates for *k* = 9 with numbers at risk. PC, principal component; PCA, PC analysis.

To evaluate the downstream utility of the synthetic data, the original and synthetic data sets were combined and clustered. Results across varying cluster numbers (*k = *2–25) are presented in Figure 7. Silhouette scores peaked at *k = *2 with a fluctuating decline and other lower peaks, notably at *k = *5, *k = *7, and *k = *9. WCSS decreased monotonically with increasing cluster numbers. Elbow points were visible at *k = *7 and *k = *9. Three-dimensional PCA visualizations and survival curves for these *k*s are shown in Figure 8.

## DISCUSSION

Although the LTx field continues to evolve, PGD remains a challenge and a risk factor for mortality.^32^ In our study, we identified 4 dynamic PGD classes with significantly different survival. Unsupervised ML revealed clear insights into important clinical features driving the clustering. We generated synthetic patient data, doubling our data set and enabling robust downstream evaluation. These complementary approaches provide a deeper understanding of PGD heterogeneity and may serve as valuable tools for risk stratification in LTx.

The first ISHLT PGD consensus guidelines, published in 2005, were revised in 2016.^2,4^ Since then, multiple studies have been conducted to validate the definition of PGD and its association with outcomes.^5,6,33-37^ In a 2010 multicenter study by Christie et al,^8^ “late PGD” at 48 and 72 h discriminated mortality better than “early PGD” at 24 h. These results resonate with our findings; persistent PGD patients had the worst 5-y survival (67%), followed by late PGD (71%). However, early PGD patients had a 77% 5-y survival. In 2013, a study by Shah et al^7^ introduced 3 classes of PGD phenotypes, only including patients with PGD3 within 72 h: “severe persistent,” “complete resolution,” “attenuation without complete resolution.” Although our persistent and early PGD phenotypes resemble classes 1 and 2, respectively, our study included all patients and introduced other classes (late and no PGD) to visualize the dynamic aspect.

The temporal aspect also aligns with previous experimental and clinical studies that suggest PGD is not a uniform process.^38-41^ Early PGD is considered to be transient, caused primarily by hydrostatic mechanisms inducing interstitial and alveolar edema.^1^ In contrast, late PGD patients have an increasing PGD grade, suggesting crosstalk between innate and adaptive immunity, resulting in endothelial-epithelial alveolar membrane injury.^42,43^ The acute injury caused by the inflammatory nature of late PGD may explain poorer survival. Persistent PGD may be considered a mixed phenotype, in which both hydrostatic mechanisms and immunity are highly active, resulting in sustained lung function impairment.

The multifactorial nature of the most discriminative features highlights the relevance of combining temporally resolved PGD data with data from different LTx stages (recipient characteristics, surgical factors, postoperative features, etc). Our unsupervised ML approach automatically determined the relevance of the 30 features for distinguishing between clusters, without any manual intervention. Postoperative chest x-rays were among the most important features affecting the clustering. These findings strengthen the current PGD grading and highlight the potential value of our temporal PGD approach.

A study by Braithwaite et al^9^ proposed an individualized framework to identify PGD risk profiles using specific donor, recipient, and intraoperative features. In a recent work by Diamond et al,^44^ they developed a PGD predictive model to assess the risks of different donor/recipient combinations. In contrast to these prediction-oriented approaches, our aim was not to predict PGD occurrence or outcome, but to explore whether unsupervised ML could provide a data-driven classification.^45^ Additionally, the most important features in our clustering (eg, preoperative ICU stay) could be used to adapt such risk assessment tools. Furthermore, by moving beyond static PGD grading and incorporating temporal patterns, we might better understand the specific risk factors for developing different phenotypes of PGD. Clinically, this transition from “grading” to “phenotyping” might provide better insights to predict whether given phenotype is more donor- or recipient-derived.

In our clustering, donor factors are among the least important features, which potentially reflects strict donor selection. Sex matching is a common practice in our clinic, likely causing donor and recipient sex to be discriminative factors. However, donor smoking is one of the most important features, especially for *k = *9, although it should be kept in mind that smoking was incompletely reported, which our ML model handled as a missing-data category.^24^ Our results are in line with the findings of Diamond et al, showing that donor smoking does not increase recipient mortality, but was found to increase PGD risk.^12^

Our study complements the work by Delen et al,^46^ the only study to our knowledge that applied *k*-means clustering in the LTx field. They used supervised ML to select features in heart transplant and LTx, followed by clustering into risk groups. In contrast, our approach avoids selecting features before clustering; instead, we applied unsupervised clustering directly to the full feature space, allowing the most informative features to emerge from the data. Delen et al tested *k = *2 to *k = *5 and chose *k = *3 based on WCSS and survival curves; we explored further options from *k = *2 to *k = *25 and determined *k = *5 and *k = *9 as optimal using combined metrics.

The unsupervised clustering approach provides a data-driven way to explore how multiple clinical factors interact, without relying on assumptions. Rather than identifying isolated risk factors, this approach highlights patterns of co-occurring features that may characterize distinct clinical profiles, which could support the development of more refined risk stratification strategies.

To address the challenges of small cohort size, we implemented a WGAN-GP model. Stabilization of the generator and critic indicated balanced learning, validating the effectiveness of the gradient penalty. The synthetic data set preserved key distributional properties of the original, both in categorical and numerical features (Figure 6). Our findings align with those of Arvanitis et al,^47^ who produced large-scale data sets using multiple methods. Metrics such as correlation matrices, as we used in our study, revealed that WGAN-GP models can generate statistically equivalent synthetic data sets in healthcare applications.^47^ Adding the synthetic data set to the original doubled our data set, providing a robust basis for clustering and evaluation.

In our research, we also used the synthetic data set for downstream evaluation. We compared *k*-means clustering results from the original patient and the combined data set. Comparing Figures 4A and 7A, consensus silhouette scores peaked at *k = *2, with a similar declining pattern and favorable clustering outcomes (*k = *5; *k = *9 or *k = *7). The WCSS for the combined data set was similar to that of the original. The Sankey diagram (Figure 7B) highlights that many patient groupings remain stable, indicating consistent subgroup formation across both the original and combined data sets. Visual cluster formation in PCA plots suggests clinical coherence among original and synthetic patient profiles. These findings, acknowledging that ML is dependent on large, high-quality data sets, demonstrate the value of synthetic data in overcoming sample-size limitations and, therefore, its potential to enhance further research in LTx.

### Limitations

This was a single-center study, which may limit generalizability. Intercenter differences could affect the overall cluster structure, possibly causing a subset of “borderline” patients near cluster boundaries to switch clusters, rather than producing entirely different phenotypes. As a follow-up, a validation study with a larger multicenter cohort is planned. Heterogeneity in the LTx population may have caused overlaps among clusters. Some features had incomplete data, whereas we used a missing-data category to overcome this issue; this may have deviated the results. Although consensus clustering and WGAN-GP methods enhance robustness, external validation on independent data sets is necessary. The binary nature of 1-hot-encoded categorical features and continuous numerical features may skew feature importance toward categorical features. Finally, using synthetic patient data raises important questions, particularly regarding reproducibility and bias amplification, highlighting the need for clear documentation, validation, and ethical oversight.

## CONCLUSIONS

In this study, temporal PGD classification reflects how the dynamic nature of PGD correlates with survival, and ML methods, such as unsupervised clustering and synthetic data generation, can determine complex interactions between clinical factors and overcome sample-size limitations inherent to transplant populations. In combination, these approaches could facilitate future efforts toward integrating multidimensional clinical data into decision-support tools.

## ACKNOWLEDGMENTS

The authors thank all members of the Leuven Lung Transplant Program for their efforts in the care of the donors and recipients. Additionally, the authors are grateful for the guidance of Marie-Francine Moens in synthetic data generation.

## Supplementary Material


