# Supplementary material for: Primary Graft Dysfunction After Lung Transplantation: A Temporal Classification and Machine Learning Clustering
Source: Transplant Direct. 2026 Jul 20;12(8):e1984. doi: 10.1097/TXD.0000000000001984 (PMC13387764; doi:10.1097/TXD.0000000000001984)

Table S1: Adherence to TRIPOD+AI reporting guidelines. This table outlines how each item from the TRIPOD-AI checklist is addressed in our study. Although the checklist is specifically made for prediction models, our study followed these guidelines for transparency. A checkmark denotes full adherence, items not applicable are marked as N/A.

Section/topic	Item	Development/evaluation*	Checklist item	Checkbox
**Title**				
Title	1	D;E	Identify the study as developing or evaluating the performance of a multivariable prediction model, the target population, and the outcome to be predicted	☑
**Abstract**				
Abstract	2	D;E	See TRIPOD+AI for Abstracts checklist	☑
**Introduction**				
Background	3a	D;E	Explain the healthcare context (including whether diagnostic or prognostic) and rationale for developing or evaluating the prediction model, including references to existing models	☑
	3b	D;E	Describe the target population and the intended purpose of the prediction model in the context of the care pathway, including its intended users (eg, healthcare professionals, patients, public)	☑
	3c	D;E	Describe any known health inequalities between sociodemographic groups	N/A
Objectives	4	D;E	Specify the study objectives, including whether the study describes the development or validation of a prediction model (or both)	☑
**Methods**				

Data	5a	D;E	Describe the sources of data separately for the development and evaluation datasets (eg, randomised trial, cohort, routine care or registry data), the rationale for using these data, and representativeness of the data	☑
	5b	D;E	Specify the dates of the collected participant data, including start and end of participant accrual; and, if applicable, end of follow-up	☑
Participants	6a	D;E	Specify key elements of the study setting (eg, primary care, secondary care, general population) including the number and location of centres	☑
	6b	D;E	Describe the eligibility criteria for study participants	☑
	6c	D;E	Give details of any treatments received, and how they were handled during model development or evaluation, if relevant	☑
Data preparation	7	D;E	Describe any data pre-processing and quality checking, including whether this was similar across relevant sociodemographic groups	N/A
Outcome	8a	D;E	Clearly define the outcome that is being predicted and the time horizon, including how and when assessed, the rationale for choosing this outcome, and whether the method of outcome assessment is consistent across sociodemographic groups	N/A
	8b	D;E	If outcome assessment requires subjective interpretation, describe the qualifications and demographic characteristics of the outcome assessors	N/A
	8c	D;E	Report any actions to blind assessment of the outcome to be predicted	N/A
Predictors	9a	D	Describe the choice of initial predictors (eg, literature, previous models, all available predictors) and any pre-selection of predictors before model building	☑

	9b	D;E	Clearly define all predictors, including how and when they were measured (and any actions to blind assessment of predictors for the outcome and other predictors)	☑
	9c	D;E	If predictor measurement requires subjective interpretation, describe the qualifications and demographic characteristics of the predictor assessors	☑
Sample size	10	D;E	Explain how the study size was arrived at (separately for development and evaluation), and justify that the study size was sufficient to answer the research question. Include details of any sample size calculation	☑
Missing data	11	D;E	Describe how missing data were handled. Provide reasons for omitting any data	☑
Analytical methods	12a	D	Describe how the data were used (eg, for development and evaluation of model performance) in the analysis, including whether the data were partitioned, considering any sample size requirements	☑
	12b	D	Depending on the type of model, describe how predictors were handled in the analyses (functional form, rescaling, transformation, or any standardisation)	☑
	12c	D	Specify the type of model, rationale, all model building steps, including any hyperparameter tuning, and method for internal validation	☑
	12d	D;E	Describe if and how any heterogeneity in estimates of model parameter values and model performance was handled and quantified across clusters (eg, hospitals, countries). See TRIPOD-Cluster for additional considerations‡	N/A

	12e	D;E	Specify all measures and plots used (and their rationale) to evaluate model performance (eg, discrimination, calibration, clinical utility) and, if relevant, to compare multiple models	☑
	12f	E	Describe any model updating (eg, recalibration) arising from the model evaluation, either overall or for particular sociodemographic groups or settings	☑
	12g	E	For model evaluation, describe how the model predictions were calculated (eg, formula, code, object, application programming interface)	☑
Class imbalance	13	D;E	If class imbalance methods were used, state why and how this was done, and any subsequent methods to recalibrate the model or the model predictions	N/A
Fairness	14	D;E	Describe any approaches that were used to address model fairness and their rationale	☑
Model output	15	D	Specify the output of the prediction model (eg, probabilities, classification). Provide details and rationale for any classification and how the thresholds were identified	☑
Training versus evaluation	16	D;E	Identify any differences between the development and evaluation data in healthcare setting, eligibility criteria, outcome, and predictors	N/A
Ethical approval	17	D;E	Name the institutional research board or ethics committee that approved the study and describe the participant informed consent or the ethics committee waiver of informed consent	☑
**Open science**				
Funding	18a	D;E	Give the source of funding and the role of the funders for the present study	☑
Conflicts of interest	18b	D;E	Declare any conflicts of interest and financial disclosures for all authors	☑
Protocol	18c	D;E	Indicate where the study protocol can be accessed or state that a protocol was not prepared	☑

Registration	18d	D;E	Provide registration information for the study, including register name and registration number, or state that the study was not registered	N/A
Data sharing	18e	D;E	Provide details of the availability of the study data	X
Code sharing	18f	D;E	Provide details of the availability of the analytical code	X
**Patient and public involvement**				
Patient and public involvement	19	D;E	Provide details of any patient and public involvement during the design, conduct, reporting, interpretation, or dissemination of the study or state no involvement	N/A
**Result**				
Participants	20a	D;E	Describe the flow of participants through the study, including the number of participants with and without the outcome and, if applicable, a summary of the follow-up time. A diagram may be helpful	☑
	20b	D;E	Report the characteristics overall and, where applicable, for each data source or setting, including the key dates, key predictors (including demographics), treatments received, sample size, number of outcome events, follow-up time, and amount of missing data. A table may be helpful. Report any differences across key demographic groups	☑
	20c	E	For model evaluation, show a comparison with the development data of the distribution of important predictors (demographics, predictors, and outcome)	☑
Model development	21	D;E	Specify the number of participants and outcome events in each analysis (eg, for model development, hyperparameter tuning, model evaluation)	☑

Model specification	22	D	Provide details of the full prediction model (eg, formula, code, object, application programming interface) to allow predictions in new individuals and to enable third party evaluation and implementation, including any restrictions to access or reuse (eg, freely available, proprietary)	X
Model performance	23a	D;E	Report model performance estimates with confidence intervals, including for any key subgroups (eg, sociodemographic). Consider plots to aid presentation	☑
	23b	D;E	If examined, report results of any heterogeneity in model performance across clusters. See TRIPOD-Cluster for additional details	☑
Model updating	24	E	Report the results from any model updating, including the updated model and subsequent performance	N/A
**Discussion**				
Interpretation	25	D;E	Give an overall interpretation of the main results, including issues of fairness in the context of the objectives and previous studies	☑
Limitations	26	D;E	Discuss any limitations of the study (such as a non-representative sample, sample size, overfitting, missing data) and their effects on any biases, statistical uncertainty, and generalisability	☑
Usability of the model in the context of current care	27a	D	Describe how poor quality or unavailable input data (eg, predictor values) should be assessed and handled when implementing the prediction model	☑
	27b	D	Specify whether users will be required to interact in the handling of the input data or use of the model, and what level of expertise is required of users	☑
	27c	D;E	Discuss any next steps for future research, with a specific view to applicability and generalisability of the model	☑

Table S2: Effect of temporal PGD classes in multivariable Cox regression on 5-year survival. HR: hazard ratio, CI: confidence interval.

	HR	95% CI	p value
**Temporal PGD class**			
No PGD *(reference)*			
Early PGD	1.24	0.73-2.11	0.49
Late PGD	1.59	0.92-2.73	0.095
Persistent PGD	1.84	1.17-2.89	**0.008**
**Donor smoking**	1.40	0.98-2.01	0.068
**Implantation time (longest of 2 lungs), per 10 minutes**	1.12	1.00-1.26	**0.048**
**ECMO bridging**	0.94	0.30-2.94	>0.9
**Recipient age**	1.04	1.02-1.07	**0.001**
**Transplant indication**			
Chronic obstructive pulmonary disease *(reference)*			
Interstitial lung disease	1.24	0.81-1.90	0.3
Cystic fibrosis	0.62	0.19-1.96	0.4
Other	1.99	0.92-4.33	0.082
**Preoperative ICU stay**	0.65	0.15-2.70	0.5
**Preoperative mechanical ventilation**	3.49	0.73-16.6	0.12

Table S3: Feature dispersion across cluster centroids for k=5 and k=9. Ranked feature-wise range across consensus cluster centroids, which shows the max and min distance between centroids, and ranked feature-wise standard deviation across consensus cluster centroids, which shows the spread of the centroids within the feature space, for *k*=5 (**panels A and B**, respectively) and *k*=9 (**panels C and D**, respectively).

**A**	**Feature**	**Range for k=5**		**B**	**Feature**	**Standard deviation for k=5**
	Chest X-ray at 48h	0.94990689			Chest X-ray at 48h	0.440168366
	Donor sex	0.921172161			Donor sex	0.422358275
	Preoperative ICU stay	0.919582891			Recipient sex	0.418687574
	Chest X-ray at 24h	0.866376812			Chest X-ray at 24h	0.415503474
	Recipient sex	0.860732601			Preoperative ICU stay	0.406315152
	Chest X-ray at 72h	0.837681159			Chest X-ray at 72h	0.385764092
	Intraoperative ECLS	0.757839721			Preoperative ventilation: no	0.325884246
	Preoperative hospital stay	0.742026266			Preoperative hospital stay	0.318498491
	Preoperative ventilation: no	0.731707317			Intraoperative ECLS	0.314285377
	Chest X-ray at 0h	0.64397197			Chest X-ray at 0h	0.295354172
	COPD	0.630125972			COPD	0.288812963
	Preoperative ventilation: invasive	0.585365854			Preoperative ventilation: invasive	0.261783568
	ECMO bridging	0.536585366			Interstitial lung disease	0.258509234
	Interstitial lung disease	0.525203252			ECMO bridging	0.238628339
	CVA	0.433898904			CVA	0.188291145
	Other cause of death	0.341753704			Other cause of death	0.126061287
	Donor smoking: no	0.270765547			Donor smoking: no	0.107637207
	Donor type	0.227026152			Donor smoking: yes	0.101771124
	Donor smoking: yes	0.224759129			Previous thoracic surgery	0.092855541
	Radiological findings: abnormal	0.207804878			Donor type	0.088993826
	Previous thoracic surgery	0.204100777			Head trauma	0.086345716
	Head trauma	0.200884481			Radiological findings: abnormal	0.079516235
	PaO_2/FiO_2 ratio at 72h	0.16371573			Radiological findings: normal	0.069752935
	Radiological findings: normal	0.156521739			PaO_2/FiO_2 ratio at 72h	0.068173986
	Preoperative ventilation: non-invasive	0.146341463			Preoperative ventilation: non-invasive	0.064159646
	PaO_2/FiO_2 ratio at 48h	0.139949187			PaO_2/FiO_2 ratio at 48h	0.056049128
	PaO_2/FiO_2 ratio at 24h	0.133907333			Radiological findings: missing	0.055427993
	Radiological findings: missing	0.129727819			PaO_2/FiO_2 ratio at 24h	0.054547751
	Recipient age	0.128029073			Recipient age	0.052057254

	Donor age	0.108311534			Total ischemia time: longest	0.047167489
	Total ischemia time: longest	0.10634786			PaO_2/FiO_2 ratio at 0h	0.044319198
	PaO_2/FiO_2 ratio at 0h	0.103913038			Donor age	0.044055726
	Cystic fibrosis	0.101715358			Cystic fibrosis	0.042992206
	Recipient BMI	0.093553773			Recipient BMI	0.038700606
	Implantation time: longest	0.076403162			Implantation time: longest	0.03514952
	Donor smoking: missing	0.062669215			Donor smoking: missing	0.029097386
	Ice storage	0.062276423			Normothermic EVLP	0.022946113
	Normothermic EVLP	0.06			Ice storage	0.022183674
	Other indication for LTx	0.043188406			Other indication for LTx	0.016385353
	Donor ventilation	0.031486281			Donor ventilation	0.015004888
	Donor PaO_2/FiO_2 ratio	0.02421416			Donor PaO_2/FiO_2 ratio	0.010589968
	Donor BMI	0.022559854			Donor BMI	0.009286194
	Controlled hypothermic	0.01942029			Controlled hypothermic	0.007655238

**C**	**Feature**	**Range for k=9**		**D**	**Feature**	**Standard deviation for k=9**
	Other cause of death	1			Chest X-ray at 48h	0.428980362
	Donor smoking: yes	1			Chest X-ray at 24h	0.413141285
	Preoperative ICU stay	1			Recipient sex	0.3919031
	Radiological findings: normal	0.97979798			Donor sex	0.384813787
	Donor smoking: no	0.972222222			Donor smoking: yes	0.378610772
	Donor sex	0.965517241			Chest X-ray at 72h	0.369913016
	Chest X-ray at 48h	0.964024481			Donor smoking: no	0.360775189
	Preoperative ventilation: no	0.9375			Preoperative ICU stay	0.325252228
	Recipient sex	0.925985605			Preoperative ventilation: no	0.311512237
	CVA	0.91954023			Other cause of death	0.301373073
	Chest X-ray at 24h	0.909090909			CVA	0.300157443
	Chest X-ray at 72h	0.860576205			Chest X-ray at 0h	0.288548253
	Intraoperative ECLS	0.790416667			Radiological findings: normal	0.26401761
	Preoperative hospital stay	0.754583333			COPD	0.250049577
	Preoperative ventilation: invasive	0.75			Preoperative ventilation: invasive	0.25
	Radiological findings: abnormal	0.746835443			Intraoperative ECLS	0.244509385
	Chest X-ray at 0h	0.693925234			Preoperative hospital stay	0.23771672
	COPD	0.691060127			Interstitial lung disease	0.230668116
	ECMO bridging	0.625			Radiological findings: abnormal	0.218102577
	Interstitial lung disease	0.605617089			ECMO bridging	0.206541419
	Donor type	0.394916911			Donor type	0.117727894
	Head trauma	0.280373832			Previous thoracic surgery	0.095625119
	Radiological findings: missing	0.278499278			Radiological findings: missing	0.094817915
	Previous thoracic surgery	0.273734177			Head trauma	0.093368503

	Preoperative ventilation: non-invasive	0.1875			Preoperative ventilation: non-invasive	0.061701826
	Recipient age	0.153431373			PaO_2/FiO_2 ratio at 72h	0.055893684
	Donor smoking: missing	0.14953271			PaO_2/FiO_2 ratio at 24h	0.051171407
	PaO_2/FiO_2 ratio at 48h	0.148890115			Donor age	0.049914536
	PaO_2/FiO_2 ratio at 72h	0.148778627			Donor smoking: missing	0.049795301
	Donor age	0.144309519			PaO_2/FiO_2 ratio at 48h	0.049201912
	PaO_2/FiO_2 ratio at 24h	0.138319864			Recipient age	0.045074912
	Total ischemia time: longest	0.121046411			Cystic fibrosis	0.041316795
	Cystic fibrosis	0.120833333			Total ischemia time: longest	0.040706488
	PaO_2/FiO_2 ratio at 0h	0.11673217			PaO_2/FiO_2 ratio at 0h	0.040046511
	Implantation time: longest	0.09837963			Recipient BMI	0.034930837
	Normothermic EVLP	0.096774194			Implantation time: longest	0.032749998
	Recipient BMI	0.096717037			Ice storage	0.030471346
	Ice storage	0.088351873			Normothermic EVLP	0.028773042
	Donor PaO_2/FiO_2 ratio	0.075666146			Donor PaO_2/FiO_2 ratio	0.022037503
	Donor ventilation	0.063652473			Donor ventilation	0.021431477
	Other indication for LTx	0.063131313			Other indication for LTx	0.019457248
	Controlled hypothermic	0.045977011			Controlled hypothermic	0.017883184
	Donor BMI	0.039498234			Donor BMI	0.010997045

Supplementary Methods

Method: K-means clustering

Preprocessing & Clustering

In this study, clustering analyses were conducted on a preprocessed dataset using an ensemble consensus approach to enhance robustness and reproducibility. First, the categorical features of all patients were transformed through one-hot encoding (meaning: turns a categorical feature into rows of 0s and 1s, with a 1 marking the chosen category of that row) and missing values were treated as an additional category. To ensure uniform scale across features and mitigate the influence of differing units, all numeric data were normalized to the [0,1] range using min-max normalization (meaning: rescales the values between 0 to 1 by subtracting the minimum and dividing by the range) and missing values were not imputed.

An ensemble clustering approach was employed to evaluate cluster stability and structure over multiple clustering runs. The number of clusters, *k*, was varied from 2 to 25. For each *k*, 1000 independent k-means runs were executed with 100 replicates per run to ensure convergence and to reduce the impact of randomly selecting the initial cluster centers, inherent to k-means. During each run, cluster quality was evaluated using the silhouette score and the within-cluster sum of squares (WCSS).

A co-association matrix was incrementally built by tracking how frequently pairs of patients were assigned to the same cluster across all runs. This co-association matrix was subsequently normalized and transformed into a dissimilarity matrix (meaning: matrix that lists how different every pair of patient is. Simplification, view each pair of patients as a cluster), which served as input for hierarchical clustering with average linkage (meaning: merges the two clusters whose average pairwise distance between

their points is smallest. Simplification, two mini clusters of two patients are combined if they are close to each other) to derive consensus cluster assignments (meaning: final cluster labels you get by combining many clusters until you end up with the desired number of clusters). At this point, every patient is assigned to one of the *k* clusters.

Analysis & Visualization

For each consensus clustering solution (*k*), performance metrics, including the silhouette score and WCSS, were computed to assess cluster compactness and separation. In addition, the centroids of the consensus clusters (meaning: “average” feature values for each final cluster) were analyzed to calculate feature-wise variance, range, and standard deviation, thereby quantifying the spread and distinctiveness of cluster centers across the feature space.

To facilitate interpretation and visualization, principal component analysis (PCA) was performed on the normalized dataset, and the first three principal components were plotted in three-dimensional scatterplots, with data points (patients) colored according to their consensus cluster assignments (**Figure 5**). To evaluate the stability of clustering solutions across different *k* values, the average Silhouette scores and WCSS of the ensemble runs, as well as the consensus metrics, were plotted as a function of *k* (**Figure 4A**). WCSS elbow inspection is used to identify the point at which additional clusters yield diminishing reductions in within-cluster dispersion, while the Silhouette coefficient provided a complementary assessment of cluster compactness and separation (Yuan & Yang, Multidisciplinary Scientific Journal, 2019, https://doi.org/10.3390/j2020016; Rousseeuw, Journal of Computational and Applied Mathematics, 1987, https://doi.org/10.1016/0377-0427(87)90125-7). To provide

insights into the transitions of observations across varying cluster solutions, a Sankey diagram was generated, visualizing the flow of patients between cluster assignments as *k* increased (**Figure 4B**). The final clustered dataset was exported to an Excel file for downstream interpretation. The k-means clustering, analysis and visualization was done in MATLAB R2024b.

Formulas

$$Silhouette\ score\ (i) = \frac{b(i) - a(i)}{max\{a(i), b(i)\}}$$

i: the current point

a(i): average distance from the current point to all other points in its own cluster.

b(i): smallest average distance from the current point to points in any other cluster (nearest other cluster).

The overall silhouette score is the mean of all individual silhouette scores.

$$WCSS = \sum_{j=1}^{k} \sum_{x \in C_j} \left\| x - \mu_j \right\|^2$$

k: the number of clusters you split the data into.

j: cluster index (1 to k).

Cj: the set of data points assigned to cluster j.

μ_j: the centroid (mean point) of cluster j.

x: a single data point that belongs to cluster Cj.

Method: GAN

A Wasserstein Generative Adversarial Network with Gradient Penalty (WGAN-GP) was employed to generate synthetic data. This architecture consisted of two neural networks (meaning: machine learning models made of connected layers that learn patterns by adjusting their connection weights from data): a generator and a critic (discriminator).

The generator was responsible for creating synthetic data from random latent noise vectors (meaning: a random set of numbers that were used as the starting point), while the critic assessed the authenticity of these synthetic samples (patients) by comparing them to real data (from the clinical dataset, after one-hot encoding and normalization). The generator is a fully connected feedforward network with three hidden layers (256, 256 and 128 units, LeakyReLU activation), followed by an output layer to match the [0, 1] scaled feature space. The critic is a similar feedforward network with hidden layers of 256, 256 and 128 units (ReLU activation), and a single linear output neuron. Unlike traditional GANs, no sigmoid is applied at the output of the critic to allow Wasserstein distance (meaning: a way to measure how different two distributions of patients are, roughly the “minimum effort” needed to transform one into the other) estimation.

The training followed the standard WGAN-GP procedure, updating the critic three times per generator update. Gradient penalty (meaning: a training term that enforces smooth, stable model behavior by penalizing overly large gradients) was computed on interpolated samples to enforce the Lipschitz constraint (meaning: forcing a model’s output to not change too fast when the input changes) required for stable Wasserstein distance approximation. Both networks were optimized using the Adam optimizer

(meaning: an optimization method that updates model weights using adaptive learning rates based on running averages of the gradients) with a learning rate of 0.0005 and β-values of 0.0 and 0.9. The latent space dimension (meaning: the number of values in the model's input noise vector) was set to 32, and models were trained for 8000 epochs (meaning: the number of times the model goes through the entire training dataset during training) with a batch size (meaning: the number of training samples the model processes at once before updating its weights) of 64.

Synthetic data were generated by sampling from a standard normal distribution and passing the vectors through the trained generator. This generating phase was repeated until 690 valid synthetic samples patients were collected. Binary columns were rounded to either 0 or 1 to restore their original discrete nature. Non-binary columns were rescaled back to their original numeric ranges using the stored minimum and maximum values from the preprocessing step.

To evaluate the similarity between the real and synthetic datasets, boxplots were produced to visually assess the distribution of each non-binary feature in both datasets (**Figure 6A**). Correlation matrices were computed and visualized as heatmaps to compare the relationships between features in real and synthetic data (**Figure 6C-E**). Additionally, for binary features, the mean values in both datasets were compared to ensure accurate representation (**Figure 6B**).

Data Augmentation with Synthetic Samples

To evaluate the impact of dataset augmentation on machine learning performance, the synthetic data generated by the WGAN-GP model were combined with the original dataset. This effectively doubled the size of the training data. The resulting merged dataset was then used as input for downstream clustering algorithms, as in the first

part of this study (**Figure 7**). The goal of this augmentation step was to demonstrate that generating synthetic data can be a viable strategy for enhancing machine learning performance, particularly when working with limited real-world samples as in lung transplantation. By ensuring that the synthetic data preserved the statistical and structural properties of the real data with the WGAN-GP, the combined dataset provided a robust basis for clustering and evaluation.

Figure S1: Histogram showing PGD grades (PGD0 to PGD3 based on ISHLT guidelines) at each timepoint from 0h to 72h after LTx.

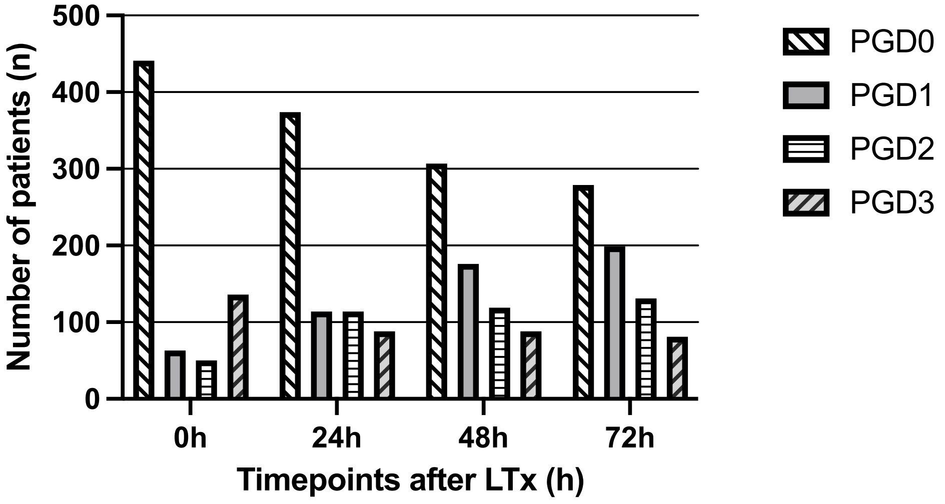

Figure S2: Generator and critic losses within the WGAN-GP model. This figure illustrates the training dynamics of a WGAN-GP over 8000 training epochs. The blue line represents the critic loss, while the orange and green line denotes the total generator loss and WGAN generator loss, respectively. Additionally, the red line shows the statistical loss by comparing the synthetic dataset with the original dataset. During the initial training phase (epochs 0–1000), both the critic and generator losses show high variability, reflecting the model's attempt to learn meaningful structure in the data. From approximately epoch 1000 onward, both loss curves stabilize, with fluctuations remaining within a bounded range. The relatively stable and opposing trend between the loss curves is a desirable characteristic in WGAN-GP training.

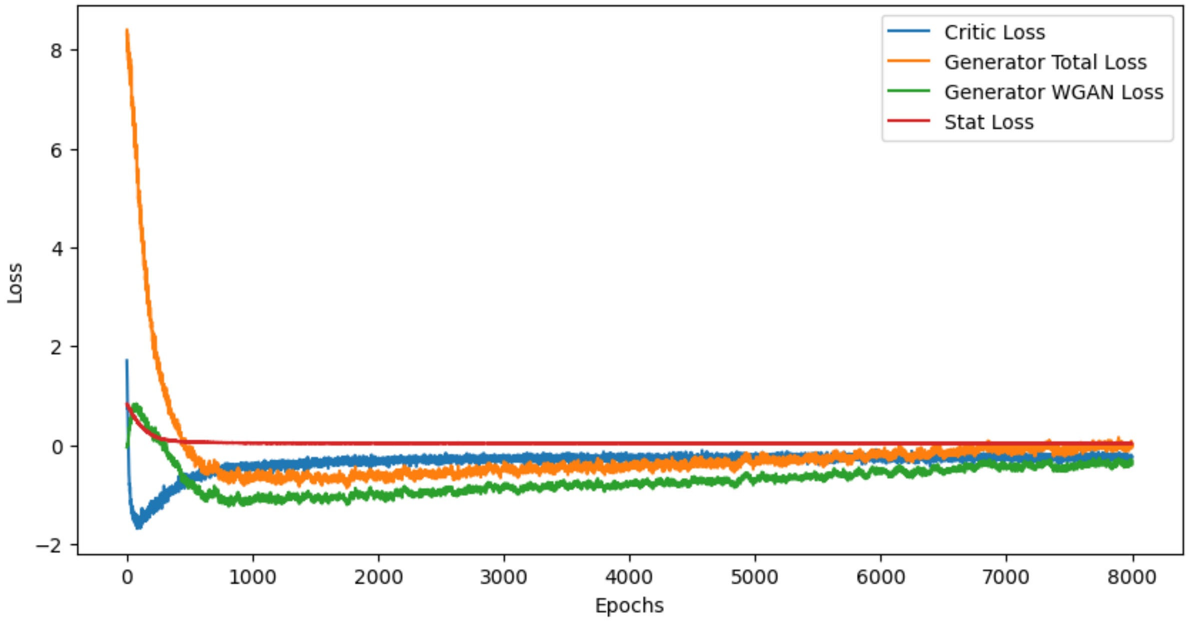

Supplement: Supplementary file 1 [file txd-12-e1984-s001.pdf]
